# Comment on: “The treatment of sarcoptic mange in wildlife: a systematic review”

**DOI:** 10.1186/s13071-020-04347-0

**Published:** 2020-09-15

**Authors:** Barbara Moroni, Marta Valldeperes, Emmanuel Serrano, Jorge Ramón López-Olvera, Santiago Lavín, Luca Rossi

**Affiliations:** 1 Dipartimento di Scienze Veterinarie, Universitá di Torino, Grugliasco, Torino Italy; 2 Wildlife Ecology & Health group (WE&H), and Serveid’Ecopatologia de Fauna Salvatge (SEFaS), Departament de Medicina i Cirurgia Animals, Universitat Autònoma de Barcelona (UAB), Bellaterra, Spain

**Keywords:** *Sarcoptes scabiei*, Wildlife diseases, Ivermectin, Drug resistance, Soil pollution

## Abstract

This letter comments on the article “The treatment of sarcoptic mange in wildlife: a systematic review” published in *Parasites & Vectors* 2019, 12:99, and discusses the limitations in the use of endectocides for scabies control in free-ranging wildlife. The ecological impact and drug resistance to ivermectin are also discussed. In our view, scabies control in free-ranging wildlife should be based preferably on population management measures, and whether to apply individual treatments to free-ranging populations should be considered very carefully and avoided where not absolutely warranted.

## Letter to the Editor

Recently, Rowe and colleagues have published an interesting review of the treatment of sarcoptic mange (hereafter “scabies”) in wildlife [1]. This review highlighted the impact of this worldwide distributed parasitic disease and pointed out the need for consensus in the implementation of effective treatment of captive and free-ranging wildlife affected by scabies.

Although we fully agree on the need for a convention on scabies control in wildlife, we would like to draw attention to the challenges and risks of implementing protocols based on pharmacological treatments in the wild. In this letter, we will set out some reasons for the incongruency of such drug-based approach.

After the systematic review of 2205 publications, Rowe et al. [1] kept 28 relevant articles reporting pharmacological effective treatments of scabies in wildlife, notably ivermectin delivered multiple times *via* subcutaneous injection. Most of these studies share a clinical treatment approach rather than population-based disease management.

Since the availability of ivermectin formulations for animal use in 1975, this drug became the most effective and safe treatment for a broad range of endo- and ectoparasites including *Sarcoptes scabiei* [2–4]. In fact, ivermectin is included amongst the election drugs to treat scabies not only in humans [5, 6] but also in domestic [7] and wild mammals [8]. This macrocyclic lactone is mainly delivered orally (e.g. in the form of drenching), pour-on or subcutaneously [2, 7] in physical or chemical restrained individuals. Based on our professional experiences and field research using ivermectin and drugs with related treatment regime methods, ivermectin treatments may be feasible for target individuals but highly challenging to unrealistic for whole populations in most instances.

Most of the studies reviewed by Rowe et al. [1] were focused on captive wildlife and only six out of the 28 papers were supposed to describe the output of ivermectin to control mange in free-ranging wildlife. Nevertheless, and after a careful reading of these articles, we noted that none of them was about the use of ivermectin to control an ongoing scabies outbreak in wild free-ranging populations. For example, the studies of Skerratt et al. [9] and Ruykys et al. [10] were largely conducted on wombats (*Vombatus ursinus* and *Lasiorhinus latifrons*, respectively) kept in captivity until their recovery. On the other hand, Kalema-Zikusoka et al. [11] worked with a group of four free-ranging gorillas (*Gorilla beringei*) habituated to human presence which facilitated their approach and treatment. Similarly, Chhangani et al. [12], treated a troop of Hanuman langurs (*Semnopithecus entellus*) living close to human habitations and religious places. Expanding urbanization and habitat loss, are leading to increasingly common human-wildlife interfaces [13], and as a result, much of the free-ranging wildlife identified as suffering from sarcoptic mange and becoming candidates for treatment might be habituated to humans to varying degrees.

Rajkovic-Janje et al. [14] focused on the use of ivermectin for endoparasite control in the wild boar. Sarcoptic mange was detected only in skin samples collected after the necropsy of four piglets. Moreover, sarcoptic mange can occur subclinically in this species [15] and others, and therefore the lack of clinical signs after the ivermectin treatment is not always a reliable indicator of recovery. Gakuya et al. [16], however, showed the outcomes of successful ivermectin treatment of endemic scabies in Thomson’s gazelles (*Eudorcas thomsonii*) and cheetahs (*Acinonyx jubatus*) from the Masai Mara National Park. The peak of scabies prevalence here ranged from 7.4% (*n* = 10 scabietic gazelles) to 28% (*n* = 2 scabietic cheetahs) affecting a small number of individuals that were captured and ear-tagged for potential re-treatment. Even though that work was representing an outbreak in free-ranging wildlife, the numbers of affected individuals are far from those recorded during scabies outbreaks in European fauna, e.g. the Northern chamois (*Rupicapra rupicapra*; *n =* 1696 affected individuals, 16.6% of the chamois population [17, 18]), or the Iberian ibex (*Capra pyrenaica*; *c.*7695 scabietic individuals, 80% of the ibex population in Sierra de Cazorla [19] and 3382 scabietic individuals, 23% of Sierra Nevada ibex population [20]).

Pharmacological treatment of mange in wild animals mostly produces individual healing, but its effects on achieving control or eradication in a population are mostly inconclusive [21]. Therefore, gathering more information on the population and environmental effects and on the consequences of massive antiparasitic treatments has been recently recommended, approaching the management of sarcoptic mange in wildlife populations from a wider ecological perspective [22]. As opposed to the individual approach revised by Rowe et al. [1], the success of scabies control in free-ranging wildlife depends on the size of the target population, scabies prevalence, and the feasibility of reaching the required percentage of the population with any specific treatment or measure [23]. Individualized pharmacological therapies, however, are desirable for vulnerable or endangered species where the complete recovery of specific individuals is decisive for species recovering (e.g. see an example for the Iberian lynx, *Lynx pardinus* [24], or for the black bear, *Ursus americanus* [25]). However, in abundant non-threatened and widespread populations with a high prevalence of scabies, it is unlikely that any individual approach would reach the necessary proportion of the population to prevent transmission and reinfection. This is even more evident for ivermectin due to the need for multiple doses to achieve a complete recovery and the total elimination of all mites from the host and the environment, although other long-acting drugs could be a better option. Nevertheless, the environmental and public health concerns of massive antiparasitic drug release in the environment would still persist [23].

While of recognized limited efficacy, selective culling of clinically affected individuals is also a common strategy in epizootic outbreak scenarios in free-ranging wildlife [26]. However, this population management measure is not free of disadvantages, such as the culling of individuals recovering from scabies [27] in detriment of the host population viability, as well as the possible objections from the public opinion in some particular species considered national icon, such as koalas (*Phascolarctos cinereus*) and wombats in Australia.

It is also important to acknowledge the potential for non-target environmental effects of mass administration of ivermectin. Avermectins are excreted during four days post-treatment and it can be detected in feces for up to 40 days post-defecation [28] and for more than one year in reindeer pastures [29]. In soil, ivermectin shows a half-life degradation between 7 and 217 days, depending on the solar radiation [3]. Once on the environment, this drug has pre-lethal consequences for dung beetles [30] and for other dung-dwelling invertebrates [3]. If the drug is delivered orally in feeding stuff, as per common practice in game ungulate populations in Spain, soil contamination and thus the potential effects of ivermectin on other terrestrial fauna, and possibly food chain effects, could be expected not only through fecal contamination but through the drug preparation itself. On the other hand, game treatment would limit venison consumption, as ivermectin withdrawal time in edible tissues may vary from 18 to 48 days depending on the administration route [7, 23, 31]. Finally, another concern about the use of ivermectin for scabies control in wildlife is the drug resistance phenomenon recently described in human scabies [32, 33] and also suspected in companion animals [34].

In line with Rowe et al. [1], little is known about the outcome of pharmacological mass treatments of scabies outbreaks in free-ranging wildlife populations. Previous reports and our own experience after decades of scabies investigation in mountain ungulates unveil that no strategy has ever unambiguously resulted in effective control of scabies in naive or endemically affected herds [23]. Instead, initial outbreak epizootics can become enzootic in successive waves as mite and host mutually adapt [35, 36]. We should also acknowledge that a range of environmental, host and pathogen factors can influence disease dynamics between enzootic, epizootic and disease-free scenarios [37]. Accordingly, whether to apply individual treatments to free-ranging populations should be considered very carefully and avoided when not absolutely warranted.

Bearing in mind the points provided here, we advocate for careful consideration of the potential limitations of the pharmacological treatment of free-ranging wildlife before the use of endectocide drugs in scabies outbreaks, considering: feasibility and efficacy, ecological impact, drug resistance, drug residues in meat (for animal and human consumption) and economics, among others. Balancing the relative merits of traditional ecological population-based management approaches to handle scabies outbreaks independent of drug-based treatments may be warranted in many free-ranging wildlife contexts. Similarly, a pragmatic assessment of whether the control can be achieved, and intervention therefore justified, should always be made.

## Data Availability

Not applicable.
